# A case of pulmonary melioidosis in Germany: a rare differential diagnosis in returning travelers from South-East Asia

**DOI:** 10.1007/s15010-024-02253-6

**Published:** 2024-04-26

**Authors:** Claudius Gottschalk, Marija Stojković, Sabine Zange, Peter Wolf, Julian A. F. Klein

**Affiliations:** 1 Department of Pneumology and Critical Care Medicine, Thoraxklinik, Heidelberg University Hospital, Heidelberg, Germany; 2 Department of Infectious Diseases and Tropical Medicine, Centre for Infectious Diseases, Heidelberg University Hospital, Heidelberg, Germany; 3 Bundeswehr Institute of Microbiology, Munich, Germany; 4 Department of Virology, Centre for Infectious Diseases, Heidelberg University Hospital, Heidelberg, Germany; 5 German Centre for Infection Research (DZIF), Heidelberg University Hospital Partner Site, Heidelberg, Germany; 6 German Centre for Infection Research (DZIF), Munich Partner Site, Munich, Germany

**Keywords:** Pulmonary melioidosis, Cavitary lung lesion, Case report, *Burkholderia pseudomallei*

## Abstract

**Background:**

Melioidosis is a bacterial infection associated with high mortality. The diagnostic approach to this rare disease in Europe is challenging, especially because pulmonary manifestation of melioidosis can mimic pulmonary tuberculosis (TB). Antibiotic therapy of melioidosis consists of an initial intensive phase of 2–8 weeks followed by an eradication therapy of 3–6 months.

**Case presentation:**

We present the case of a 46-year-old female patient with pulmonary melioidosis in Germany. The patient showed chronic cough, a pulmonary mass and a cavitary lesion, which led to the initial suspicion of pulmonary TB. Melioidosis was considered due to a long-term stay in Thailand with recurrent exposure to rice fields.

**Results:**

Microbiologic results were negative for TB. Histopathology of an endobronchial tumor showed marked chronic granulation tissue and fibrinous inflammation. Melioidosis was diagnosed via polymerase chain reaction by detection of Burkholderia pseudomallei/mallei target from mediastinal lymph-node tissue.

**Conclusion:**

This case report emphasizes that melioidosis is an important differential diagnosis in patients with suspected pulmonary tuberculosis and recent travel to South-East Asia.

## Presentation of the case

In September 2021, a 46-year-old female patient was admitted to hospital for the evaluation of a pulmonary mass of unknown origin.

The patient had been well until 4 weeks before admission, when dry cough with chest pain and mild sore throat developed.

**Fig. 1:**
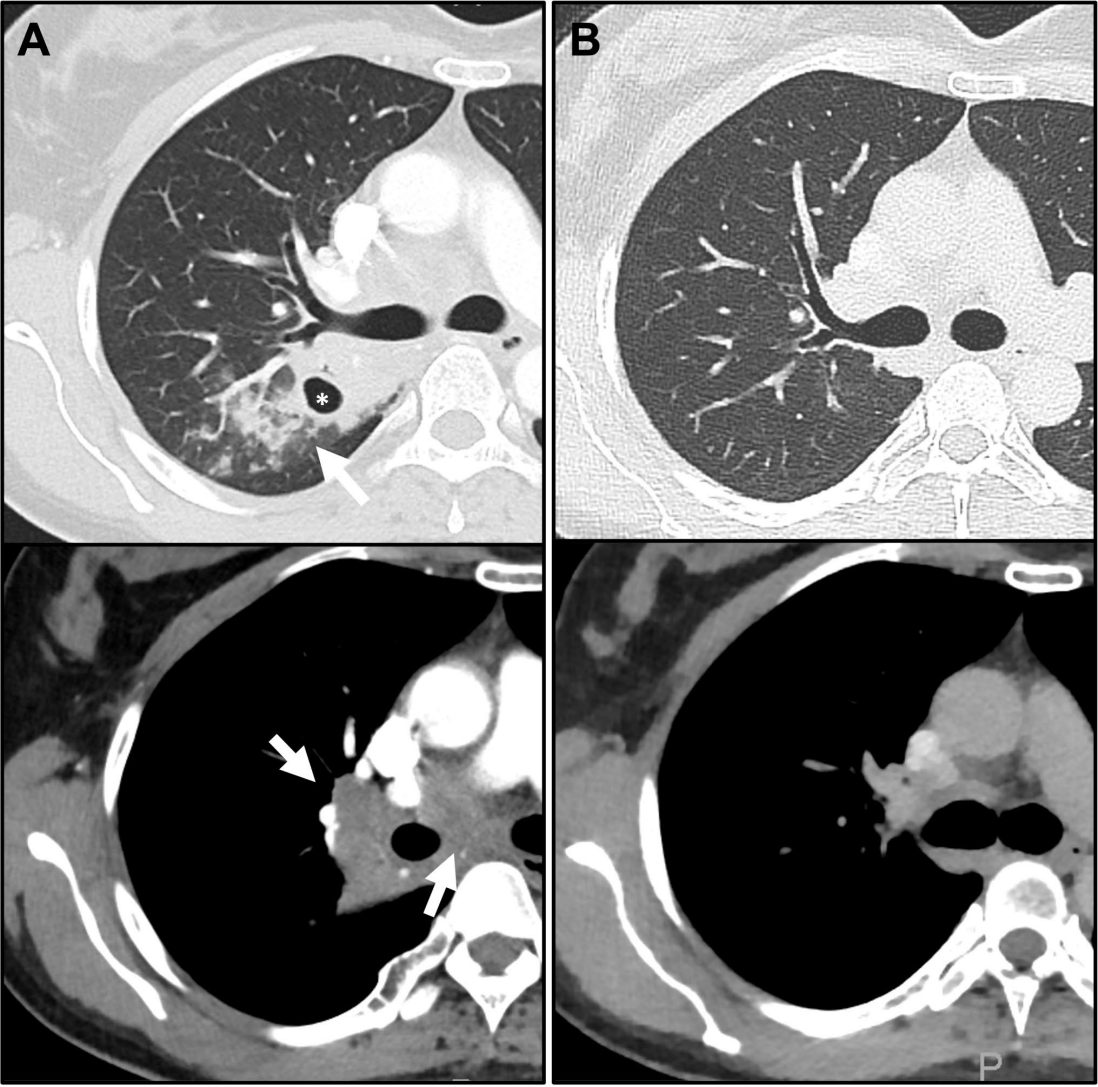
Computed tomography at baseline **(A)** and follow-up **(B)** in lung window (upper images) and soft-tissue window (lower images). A shows pulmonary infiltration in the right upper lobe with predominance of bronchocentric consolidations (upper image, arrow) and evidence of intralesional pulmonary cavitation (upper image, asterisk). There is coalescing adenopathy at the right hilum and infracarinary mediastinum (lower image, arrows). Follow-up imaging after 8 months **(B)** shows complete resolution of pulmonary infiltrates and adenopathy. (courtesy of Mathias Beyer, MD, Radiologie Lampertheim for A)

Symptomatic treatment with inhaled corticosteroids and a beta-2-agonist prior to admission was unsuccessful. Chest X-ray and a consecutive computed tomography (CT) of the chest revealed a large soft-tissue mass (diameter 5.3 × 4.6 cm) right centrally, involving the mediastinum and patchy-confluent opacities in the right upper lobe of the lung with a circumscribed cavitation (diameter 1.2 cm) (Fig. 1).

Radiologic differential diagnostic considerations included malignancies, such as bronchial carcinoma or mediastinal lymphoma, as well as pulmonary tuberculosis (TB) and other infections of the lung. The clinical history revealed that the complaints had begun three weeks after returning from a visit to Thailand. The patient had been living in Germany for the last 27 years and was born in Thailand, where she had spent 6 months visiting relatives at the countryside in northeastern Thailand (district of Somdet, Kalasin province). No one of her family experienced similar symptoms. She had no history of illness and had no known allergies. The patient did not report diabetes mellitus. There was no smoking history and she did not drink alcohol or use illicit drugs. She had two adult children, lived in a relationship, and worked in a market garden.

The laboratory results from the day of admission are shown in Table 1. Three sputum samples were preserved for mycobacterial examination including cultures.

**Table 1:** Laboratory results

Variable	On first admission	Reference range in this hospital
Hemoglobin (g/dl)	12.1	12.0–15.0
Hematocrit (%)	36.7	36–47
Erythrocytes (per nl)	4.9	4–5.2
Hemoglobin (g/dl)	12.1	12–15
Hematocrit (l/l)	0.367	0.36–0.47
Thrombocytes (per nl)	493	150–440
Leukocytes (per nl)	8.82	4–10
Differential blood count		
Neutrophils (per nl)	6.47	1.8–7.7
Lymphocytes (per nl)	1.47	1.0–4.8
Monocytes (per nl)	0.53	0.2–0.8
Eosinophils (per nl)	0.21	< 0.5
Basophils (per nl)	0.02	< 0.2
Sodium (mmol/l)	137	135–146
Potassium (mmol/l)	4.74	3.4–4.8
Calcium (mmol/l)	2.48	2.11–2.59
Chloride (mmol/l)	102	98–111
Creatinine (mg/dl)	0.67	0.5–0.9
Urea (mg/dl)	18	< 45
Aspartate transaminase (U/l)	22	< 37
Alanine transaminase (U/l)	16	< 35
Alkaline phosphatase (U/l)	71	55–105
Gamma-glutamyltransferase (U/l)	24	< 40
Lactate dehydrogenase (LDH) (U/l)	189	<308
C-reactive protein (mg/l)	12.9	< 5
International normalized ratio (INR)	1.06	< 1.2
Activated partial thromboplastin time (aPTT) (s)	24.9	< 35
Capillary blood gas analysis while breathing ambient air		
pH	7.41	7.37–7.45
pO_2_ (mmHg)	85.0	
pCO_2_ (mmHg)	41.7	35–45
Base excess (mmol/l)	1.9	< 5.0
Bicarbonate (mmol/l)	26.1	

A bronchoscopy revealed a tumor in the right main bronchus as well as enlarged and hyperechoic mediastinal and hilar lymph nodes. Biopsy samples and transbronchial needle aspirations (TBNA) were sent for microbiological and histologic examination. Bronchial washings were sent for microbiological examination.

Cultures of bronchial fluid showed no growth of bacteria after 48 h of incubation. Microbiologic and histopathological examinations of all specimens including native lymph node aspirates were negative for Mycobacterium tuberculosis, including acid-fast stain and polymerase chain reaction (PCR). Histopathologic evaluation suggested an infectious genesis but did not lead to a certain diagnosis: Specimen of the right-sided endobronchial tumor showed respiratory mucosa with marked chronic granulation tissue and fibrinous inflammation. Samples of lymph nodes showed numerous epithelioid cells and neutrophil granulocytes.

Surprisingly, our initial main differential diagnosis of TB was not confirmed. However, mycobacterial cultures were not finished at this time. Since another bacterial infection could not be ruled out, an oral, calculated antibiotic therapy with amoxicillin–clavulanic acid and clindamycin was started. The patient was discharged. In view of her recent stay in Thailand and the unexplained lung condition, a consultation at an outpatient Division of Infectious Diseases and Tropical Medicine was arranged.

After 12 days of the antibiotic treatment, the patient reported a good general constitution without any fever episodes, but she described slight pain on the right flank. The cough had abated. On enquiry, she reported recurring stays in rice fields in rural areas of Thailand in June and July. She had been helping her family with the rice harvest. Based on the patient’s history, clinical and radiologic findings, pulmonary melioidosis was considered the most likely differential diagnosis. Retroactively, blood samples and the three paraffin-embedded lymph node samples were sent to an external specialized laboratory. For serologic testing, *Burkholderia pseudomallei* (*B. pseudomallei*) LPS Type A Immunoglobulin G immunoblot was conducted from the patient’s serum. This qualitative test assay showed a positive result. PCR in one of three lymph node samples was positive for *Burkholderia-mallei/pseudomallei* DNA (target gene: *fliC*). Due to the low specific nucleic acid content in the sample, the differentiation between *B. mallei* and *B. pseudomallei* was not successful. Regarding the travel history, the clinical presentation and the serological result, we concluded that *B. pseudomallei* is the causative agent. Sputum and bronchial fluid cultures were negative for mycobacteria.

Therefore, the diagnosis of pulmonary melioidosis was made. The patient was readmitted to start a 14-day course of intravenous ceftazidime. This therapy was well tolerated.

After discharge, the patient felt healthy, was afebrile and gaining weight. Antibiotic therapy was continued with oral trimethoprim/sulfamethoxazole (80 mg/400 mg 3 tablets every 12 h) as an eradication therapy. Nine days later, she developed a pruritic allergic exanthema, most likely due to co-trimoxazol. We converted therapy to a second line regime with amoxicillin–clavulanic acid (500 mg/125 mg two tablets every 8 h). This was well tolerated and exanthema abated. Initially, the therapy term was planned for 3 months, but was extended to 5 months, based on the modification to a second line therapy. Close laboratory monitoring did not show any liver enzyme elevation or kidney function impairment. During the therapy, the patient complained several times of intermittent thoracal pressure and pain with radiation to the right thorax, while her general constitution was normal. She negated episodes of fever or dyspnea during therapy. An x-ray of the chest was inconspicuous. After the antibiotic treatment was finished in April 2022, a chest CT showed complete disappearance of the lung cavities. Furthermore, the opacities in the right upper lobe and the soft tissue surrounding the right main bronchus had reduced. Thirteen months after completing the therapy, the patient felt well and had no complaints. The intermittent chest pain had abated. A further CT of the chest in early 2023 did not show any changes, compared to the previous scan. No new lesions occurred.

## Discussion

We report the case of a 46-year-old female patient in Germany with pulmonary melioidosis after recurrent exposure to rice fields in Thailand.

Melioidosis is a multifaceted infection caused by the gram-negative bacterium *Burkholderia pseudomallei*, that is well known to inhabit soil and surface water in endemic regions (South-East Asia and northern Australia). As estimated in a modeling study 2016, the worldwide incidence of melioidosis in humans is approximately 165.000 per year, of which 89.000 (54%) cases are lethal [1]. Since the disease is not notifiable in most European countries including Germany, there is no systematic surveillance of case numbers. Data on cases in Europe are limited to case reports [2, 3].

The most important risk factor to be mentioned is the presence of diabetes mellitus, which increases the risk for melioidosis by 12 times [5]. We did not screen for diabetes mellitus in our patient, since she did not have signs of metabolic syndrome and had no family history of diabetes mellitus. Further, predisposing risk factors include exposure to soil or water (especially during rainy season), male sex, age over 45 years, harmful alcohol consumption, chronic disease of liver, lung and kidney, and immunosuppression [5, 7].

The range of clinical presentation is variable in terms of disease severity, time course and organ involvement. Melioidosis does not only occur as pneumonia (40–60%) but may also present as localized infection of skin and soft tissue (13–24%), urinary tract system (14–28%), musculoskeletal system (4–14%), central nervous system (1–5%) or even as bacteremia without evident focus (10%) [4, 5]. Most cases of melioidosis present with an acute illness, but 10–15% of patients develop a chronic disease, defined by a symptomatic lasting longer than 2 months [6]. These broad characteristics and the common occurrence of non-specific symptoms gave the disease the designation ‘the great mimicker’ [5]. Suspicion of melioidosis should be raised in patients with sepsis, pneumonia, or abscesses and a history of travel to or immigration from an endemic region [6].

Our patient initially presented with an acute but persistent dry cough, palpitation and an unspecific whole-body pain. Regarding clinical signs and radiologic findings, the differential diagnosis included infections, like pulmonary TB, hematological neoplasia (e.g., mediastinal lymphoma) and solid tumors (e.g., bronchial carcinoma). Malignant lymphoma was considered unlikely, since the complete blood count and lactate dehydrogenase were normal. Eventually, lymphoma was ruled out by histopathology of the enlarged lymph nodes. The young age of our patient and the absence of pulmonary noxae favored pulmonary TB over bronchial carcinoma. Furthermore, histopathology did not reveal any malignant cells. Other infectious etiologies include fungal infections such as invasive aspergillosis. This condition mainly occurs in the setting of immunosuppression [8], which was not present in our patient.

Based on the symptoms and radiologic findings, pulmonary TB was the initial main differential diagnosis. Unexpectedly, microscopy and PCR for TB were negative in our case. Pulmonary melioidosis is often misdiagnosed as pulmonary TB [9–11]. Both entities share the radiologic features of upper lobe infiltration, cavitation and abscess formation [12]. The coincident presence of significant pulmonary pathology and yet repeated negative diagnostic evaluation for tuberculosis should prompt further differential diagnostic considerations. In this situation, a step-up approach of clinical reasoning with a careful re-taking of the patient’s history is highly warranted. In our case, a thorough medical history revealed that the patient had spent time in rice fields during the rainy season, which guided us to the suspicion of melioidosis.

When Melioidosis is suspected, blood cultures, urine, and throat-swab specimens should be obtained. Furthermore, sputum and samples from sites of manifestation (e.g., pus from abscesses, cerebrospinal fluid, or deeper respiratory samples) should be collected [6]. Given its limited sensitivity and specificity, serologic testing plays a minor role in the diagnostic workup of melioidosis, especially because it does not allow differentiation between acute or past infection [5, 13, 14].

In our case, diagnosis was confirmed by a positive *B. pseudomallei*/*B. mallei* PCR from lymph node tissue. Blood cultures were not taken, because the main differential diagnosis were TB and malignancy. However, when the suspicion of melioidosis developed, the patient had already received an oral antibiotic therapy with amoxicillin–clavulanic acid, which could have led to false-negative culture results. Several specimens of bronchial fluid were obtained during bronchoscopy, but bacterial cultures did not yield *Burkholderia* growth. In general, optimal growth conditions for *Burkholderia pseudomallei* do not differ from standard culture conditions, but should include *Burkholderia* specific selective culture media like *Burkholderia cepacia* agar as well as Ashdown’s agar and the incubation period should be extended to 5 days [5, 15]. Therefore, the short-incubation time of 48 h might explain the negative cultures in our case, as melioidosis was not suspected at the time of sample collection. In similar cases, described before, initial cultures were also negative and the agent was cultivated in one case only from bronchoalveolar fluid using Ashdown’s agar after 72 h of incubation [16] and in a second case only from lymph node aspirates [9]. Whenever there is a suspicion of melioidosis, early notification of the microbiologic laboratory staff is crucial for correct precautions, extended incubation period and a rapid detection of the hazard group 3 pathogen *Burkholderia pseudomallei* [5].

The antibiotic treatment of melioidosis consists of an initial intensive therapy with ceftazidime or meropenem, followed by an eradication therapy with trimethoprim/sulfamethoxazole or amoxicillin–clavulanic acid [5]. Treatment duration depends on disease severity and organ involvement. Eradication therapy is given for at least 3 months [17, 18]. Extending the initial intensive phase to four weeks is recommended when specific complicating factors are present, e.g., multi-lobar pneumonia, positive blood cultures or ICU admission [7, 18]. Our patient received a 2-week course of intravenous ceftazidime, followed by an eradication therapy with amoxicillin–clavulanic acid of 5 months. Despite multilocular pulmonary manifestation and mediastinal lymphadenopathy, we did not extend initial intensive therapy, since our patient had already received an oral, calculated therapy with amoxicillin–clavulanic acid and clindamycin over 4 weeks prior to the final diagnosis.

## Conclusion

In Central Europe, melioidosis is a rare diagnosis and occurs almost exclusively in returning travelers. Awareness of this infection is crucial for clinicians outside endemic or high incidence regions, especially for those treating refugees and returning travelers. The combination of pulmonary lesions and a history of a previous stay in rural areas of South-East Asia or northern Australia should guide physicians to include melioidosis in their differential diagnosis, especially in case of negative diagnostic results for tuberculosis. This case report emphasizes the importance of conducting a detailed patient history and exposure assessment, when investigating a patient for infectious diseases.

## Data Availability

The data used in this study cannot be accessed or made available for external use, due to data regulations. The patient’s data were anonymized and will not be provided to any third party.

## Declarations

### Concern to participate

The patient has given her written consent to the publication of this case.

### Concern to publish

The patient has given her written consent to the publication of this case.
